# Withdrawal users' experiences of and attitudes to contraceptive methods: a study from Eastern district of Tehran, Iran

**DOI:** 10.1186/1471-2458-10-779

**Published:** 2010-12-22

**Authors:** Parvin Rahnama, Alireza Hidarnia, Farkhondeh Amin Shokravi, Anoushiravan Kazemnejad, Zeinab Ghazanfari, Ali Montazeri

**Affiliations:** 1 Department of Health Education, Faculty of Medicine, Tarbiat Modares University, Tehran, Iran; 2 Department of Midwifery, Shahed University, Tehran, Iran; 3 Department of Biostatistics, Faculty of Medicine, Tarbiat Moderes University, Tehran, Iran; 4 Department of Public Health, Ilam University of Medical Sciences, Ilam, Iran; 5 Department of Mental Health, Iranian Institute for Health Sciences Research, ACECR, Tehran, Iran

## Abstract

**Background:**

The aim of this study was to explore withdrawal users' experiences of and attitudes to contraceptive methods in Tehran, Iran.

**Methods:**

This was a cross-sectional study. A sample of women attending a health care center in Tehran, Iran was entered into the study. To collect data structured interviews were conducted.

**Results:**

In all 300 women who were using withdrawal took part in the study. Of these, 210 women (70%) indicated that they were used modern contraceptive methods previously. The mean duration for withdrawal use was 6.5 (SD = 4.9) and for modern contraceptive it was 2.3 (SD = 2.9) years. The most common reasons for using withdrawal were: no cost involvement, did not need medical advice, having fewer side effects and easier to use than other methods. The main obstacles to use modern contraceptives were: health concerns, fear of side effects, misinformation, lack of confidence and sexual dissatisfaction.

**Conclusion:**

The study results suggest that withdrawal users carry misconceptions about modern contraception and exaggerate its related health problems. Indeed these are important issues for the understanding of attitudes and experiences of women in Iran. The findings from this study might be essential for making evidence-based policy decisions, and for planning, monitoring and evaluating reproductive health programs in Iran and elsewhere.

## Background

Withdrawal and other traditional methods of contraception are still a method of choice for family planning in a number of developing countries such as Iran, although modern methods for family planning are easily available. Withdrawal is known to be associated with high rates of unintended pregnancy, and in turn it is associated with adverse effects including delayed prenatal care, pre-maturity and low birth weight [1,2]. Studies have shown that choosing a method of contraception and using it effectively is a complex issue influenced by more than the simple availability of information [3].

According to the Iranian Demographic Health Survey (IDHS) 17.8% of couples are using withdrawals, even though the national family planning program does not encourage this method [4]. In Iran family planning services and primary health care units provide information on contraceptive methods to individuals or couples, and these units offer contraceptives free of charge. Family planning midwives carry out counseling, intrauterine contraceptive device (IUCD) insertion and distribution of oral contraceptives. Condoms, oral contraceptives, emergency pill, injections are available all over the country. In addition, private practice gynecologists and general practitioners, as well as midwives at maternity consultations in hospitals, are among those who provide information about contraceptive methods.

Previous studies from Iran mostly focused on demographic characteristics of women who used withdrawal [5,6]. For instance a study showed that withdrawal users were younger, well educated and from urban areas [6]. However, despite the importance of these studies, they have been unable to detect the effects of other variables on choice of withdrawal as a birth control method. It is argued that it is crucial for the health care providers to assess women's attitude about family planning prior to educating them and providing them with contraceptive services [7]. Thus, the aim of this study was to investigate the reasons for using withdrawal by Iranian women and to gain a better understanding of their experiences of and attitudes to contraceptive methods. In addition, we thought the findings from this study might provide baseline information in order to help to design an educational program for use by the Iranian health care system and perhaps in other developing countries.

## Methods

### Design and participants

This was a cross-sectional study carried out in Tehran, Iran during April to September 2009. Participants were recruited from women attending five family planning clinics of public health services in the Eastern district of Tehran, Iran (the area serves as the training area for the Iran University of Medical Sciences). Interviews were conducted at certain days of the week in each clinic. Weekdays for interviews were selected randomly. Criteria for inclusion were: women aged 18-49 years, current withdrawal users, married, sexually active, and did not intend to be pregnant.

### Questionnaire

A structured questionnaire was designed in order to collect data. The questioner contained 31 items derived from the literature [8,9]. It was consisted of three parts: (i) socio-demographic characteristics including age, education, employment status, and women's reproductive health background, (ii) reasons why women were using withdrawal method and, (iii) questions about experiences of and attitudes to the contraceptive methods [Additional file 1].

### Analysis

Descriptive analyses were carried out to explore the data. Statistical procedure included chi square test for categorical data to examine the relationships between dependent and independent variables. Reasons for using withdrawal method was considered as dependent variable and age, education, employment, time since marriage and numbers of children were considered as independent variables. The SPSS version 16.0 was used to analyze the data.

### Ethics

Approval for the study was obtained from the Office for Protection of Research Subjects in Tarbiat Modares University. An oral informed consent was obtained from each participant prior to the study commence ensuring them that their identities will remain confidential.

## Results

### Sociodemographic characteristics of the study sample

In all 307 women were approached and 300 agreed to interviewed, giving a response rate of 97.7%. The mean age of participants was 31.4 (SD = 6.6) years. The mean duration of marriage was 10.0 (SD = 6.0) years. One out of four women reported that they work outside the home for financial remuneration. Reproductive history showed that nearly one out of four women had 3 or more than three children (25.7%). Overall 86 women (28.7%) reported having had at least one unwanted pregnancy. Of these, 32 women (37.2%) said that they were practicing withdrawal during unwanted pregnancy. One out of five women reported that they underwent induced abortion and they terminated pregnancy because it was unwanted. More than half (52.9%) said that they practicing withdrawal (Table 1).

**Table 1:** The characteristics of study sample (n = 300 withdrawal users)

		Frequency	%
**Age**			

	< 35	200	66.7

	≥ 35	100	33.3

	Mean (SD)	31.4 (6.6)	

**Education**			

	Primary	61	20.3

	Secondary	154	51.3

	Higher	85	28.3

**Employment status**			

	Housewife	226	75.3

	Employed	74	24.7

**Time since marriage (years)**			

	10 <	161	53.7

	10 ≥	139	46.3

	Mean (SD)	10.0 (6.0)	

**Number of children**			

	0	16	5.3

	1	89	29.7

	2	118	39.3

	3 ≥	77	25.6

**Experience of unwanted pregnancy**			

	Yes	86	28.7

	No	214	71.3

**Contraceptive methods used while experienced unwanted pregnancy **(n = 86)			

	None	23	26.7

	Withdrawal	32	37.2

	OCP*	14	16.3

	IUCD**	6	7.0

	Injection	1	1.2

	Condom	10	11.6

**Results of unwanted pregnancy (n = 86)**			

	Delivery	55	64.0

	Spontaneous abortion	13	15.1

	Induced abortion	17	19.7

	Not responded	1	1.2

**Contraceptive methods used before underwent induced abortion (n = 17)**			

	Withdrawal	9	52.9

	Condom	4	23.5

	None	3	17.6

	Pill	1	5.9

* OCP: oral contraceptive

** IUCD: intrauterine contraceptive device

### Attitudes toward contraceptive methods

There were several different attitudes towards contraceptive methods. These are presented in Table 2. In summary concerns about oral pills were nervousness (79.3%), weight gain (74.7%), and irregular bleeding (72.7%). Negative attitudes towards intrauterine device (IUCD) were irregular bleeding (57.0%), infection (34.3%), and pain (24.7%). Concerns related to condom and injection use were worries about unwanted pregnancy (37.7%) and severe irregular bleeding (24.0%), respectively.

**Table 2:** Women's (current withdrawal users') attitudes toward modern contraceptive methods (n = 300)*

Methods		Frequency	%
**OCP**			

	Nervousness (mood change)	238	79.3

	Weight gain	224	74.7

	Irregular bleeding	218	72.7

	Hirsutism	154	51.3

	Might become infertile	108	36.0

	Nausea	94	31.3

	Might get pregnant	80	26.7

**IUCD**			

	Irregular bleeding	171	57.0

	Infection	103	34.3

	Pain	74	24.7

	Translocation to other parts of the body	63	21.0

	Might get pregnant	68	22.7

**Condom**			

	Might get pregnant	113	37.7

	Husband's dislike	101	33.7

	Sexual enjoyment	68	22.7

	Difficult to use	44	14.7

**Injection**			

	Irregular bleeding	72	24.0

	Amenorrhea	58	19.3

	Weight gain	47	15.7

	Might get pregnant	12	4.0

* Since women could choose several response categories, frequencies and percentages are exceeded the study sample size and 100%.

### Previous experiences of using contraceptive methods

Overall 210 women (70%) indicated that they were using modern contraceptives previously. These were: OCP (n = 111), IUCD (n = 46), condom (n = 44), or injection (n = 9). The most common reasons for stopping contraceptive methods were as follows: 36.9% stopped taking pills because of its side effect, 32.6% stopped using IUCD due to health problems, 36.4% gave up using condom because of their husbands' dislike, and finally 44.4% stopped injection because they thought they might get pregnant. Table 3 shows the results.

**Table 3:** Women's (current withdrawal users') reasons for stopping modern contraceptive methods (n = 210)


		**Frequency**	**%**

**Experience of modern contraceptive use**			

	Yes	210	70

	No	90	30

**Contraceptive methods used before withdrawal **(n = 210)			

	OCP	111	52.9

	IUCD	46	21.9

	Condom	44	20.9

	Injection	9	4.3

OCP (n = 111)			

	Side effects	41	36.9

	Difficult to use	39	35.1

	Thought might get pregnant	19	17.1

	Willingness to be pregnant	8	7.2

	Unwillingness of their husbands	4	3.6

IUCD (n = 46)			

	Health problems	15	32.6

	Thought might get pregnant	14	30.4

	Willingness to be pregnant	8	17.4

	Refer to health care services	6	13.0

	Unwillingness of their husbands	3	6.5

Condom (n = 44)			

	Unwillingness of their husbands	16	36.4

	Thought might get pregnant	10	22.7

	Difficult to use	10	22.7

	Difficult to access	8	18.2

Injection (n = 9)			

	Thought might get pregnant	4	44.4

	Health problems	3	33.3

	Unwillingness of their husbands	2	22.2

### Experiences of and attitudes to contraceptive methods

Seventy-two percent of women (n = 216) indicated that they can talk to their spouses about contraception use. Only 34% of the women knew about emergency contraception. Nearly half of the women correctly knew the fertile period of their menstrual cycle. Just over a half of them reported that they were afraid of conceiving while they were using withdrawal method (Table 4).

**Table 4:** Women's attitudes and practice towards withdrawal method


		**Frequency**	%

**Women were pleased with this method**			

	Yes	199	66.3

	No	101	33.7

**Husbands were pleased with this method**			

	Yes	190	63.3

	No	74	24.7

	Unknown	36	12.0

**Decreased sexual enjoyment while using this method (women themselves)**			

	Yes	102	34.0

	No	148	49.3

	Unknown	50	16.7

**Decreased sexual enjoyment while using this method (women reporting on behalf of their husbands)**			

	Yes	126	42.0

	No	142	47.3

	Unknown	32	10.7

**Discussion on family planning**			

	Yes	216	72.0

	No	84	28.0

**Who makes the decision about using contraceptives in your family?**			

	Myself	39	13.0

	My Husband	45	15.0

	Myself and my husband together	216	72.0

**Fear of becoming pregnant while using this method**			

	Yes	187	62.3

	No	113	37.7

**Familiar with emergency contraception**			

	Yes	103	34.3

	No	197	65.7

**Knew their period of ovulation**			

	Yes	154	51.3

	No	146	48.7

**Use of condom during their period of ovulation**			

	Yes	43	14.3

	No	257	85.7

### Main reasons for using withdrawal method

Women indicated several reasons for using withdrawal. Most important reasons were: availability without any charge (89%), followed by having fewer side effects (80.7%) and easier to use (70.3%). The findings are summarized in Table 5.

**Table 5:** Main reasons for using withdrawal method by women with and without children (n = 300)*


**Number of children**	**None**		≥ **1**	

	**Frequency**	**%**	**Frequency**	**%**

Available without any charge	16	100	251	88.3

Fewer side-effects than other methods	10	62.5	232	81.6

Easier to use	15	93.8	196	69.0

No need for counseling with a health care provider	15	93.8	187	65.8

Husband's preference	12	75.0	152	53.5

Fear of using another method	6	37.5	145	51.0

Suggesting by a health care provider	2	12.5	41	14.4

Duration of withdrawal use (mean year, SD)	3.3 (2.2)		6.6 (4.9)	

* Since women could choose several response categories, frequencies and percentages are exceeded the study sample size and 100%.

When association between these main reasons and the respondents' age, employment status, time since marriage and number of children were studied, the results indicted that there were no significant associations between these independent variables and the main reasons for using withdrawal method. In addition, there was no significant association between educational status and two reasons stated by women [availability without any charge (P = 0.1) and easier to use (P = 0.1)]. However, there was a significant relationship between educational status and the statement that withdrawal imposes fewer side effects than modern contraception methods (P < 0.001). The results are shown in Table 6.

**Table 6:** Association between the three most important reasons to use withdrawal and women's characteristics


	**Without any charge**		**Fewer side-effects**		**Easier to use**	

	***Yes***	***No***	***Yes***	***No***	***Yes***	***No***

	**No. (%)**	**No (%)**	**No. (%)**	**No. (%)**	**No. (%)**	**No. (%)**

**Age**						

**35 >**	180 (67.4)	20 (60.6)	163 (67.4)	37 (63.8)	137 (64.9)	63 (70.8)

≥ 35	87 (32.6)	13 (39.4)	79 (32.6)	21 (36.2)	74 (35.1)	26 (29.2)

*Test for significance*	df = 1 χ2 = 0.61 P = 0.4		df = 1 χ2 = 0.26 P = 0.6		df = 1 χ2 = 0.96 P = 0.3	

**Education**						

Primary	51 (19.1)	10 (30.3)	38 (15.7)	23 (39.7)	41 (19.4)	20 (22.5)

Secondary	136 (50.9)	18 (54.5)	128 (52.9)	26 (44.8)	103 (48.8)	51 (57.3)

Higher	80 (30)	5 (15.2)	76 (31.4)	9 (15.5)	67 (31.8)	18 (20.2)

*Test for significance*	df = 2 χ2 = 4.16 P = 0.1		df = 2 χ2 = 17.96 P < 0.001		df = 2 χ2 = 4.10 P = 0.1	

**Employment**						

Housewife	200 (74.9)	26 (78.8)	177 (73.1)	49 (84.5)	156 (73.9)	70 (78.7)

Employed	67 (25.1)	7 (21.2)	65 (26.9)	9 (15.5)	55 (26.1)	19 (21.3)

*Test for significance*	df = 1 χ2 = 0.23 P = 0.6		df = 1 χ2 = 3.23 P = 0.07		df = 1 χ2 = 0.75 P = 0.3	

**Time since marriage (years)**						

10 <	148 (55.4)	13 (30.4)	128 (52.9)	33 (56.9)	114 (54.0)	47 (52.8)

10≥	119 (44.6)	20 (60.6)	114 (47.1)	25 (43.1)	97 (46.0)	42 (47.2)

	df = 1 χ2 = 3.03 P = 0.08		df = 1 χ2 = 0.30 P = 0.5		df = 1 χ2 = 0.03 P = 0.8	

**Number of children**						

0	16 (6.0)	0 (0)	10 (4.1)	6 (10.3)	15 (7.1)	1 (1.1)

1	81 (30.3)	8 (24.2)	72 (29.8)	17 (29.3)	63 (29.9)	26 (29.2)

2	106 (39.7)	12 (36.4)	102 (42.1)	16 (27.6)	82 (38.9)	36 (40.4)

≥ 3	64 (24.0)	13 (39.4)	58 (24.0)	19 (32.8)	51 (24.1)	26 (29.2)

*Test for significance*	df = 3 χ2 = 5.15 P = 0.1		df = 3 χ2 = 7.32 P = 0.06		df = 3 χ2 = 4.87 P = 0.1	

## Discussion

The findings of this descriptive study indicated that there are various obstacles to modern contraceptive use among withdrawal users. The main factors were, health concerns and fear of side effects, misinformation related to modern contraception, lack of confidence in modern methods, dissatisfaction with sexual sensation, and unwillingness of their husbands. Surprisingly religious factors were not noted by women in our study to be a reason for inability to choose effective methods.

We found that the most common reasons for using withdrawal were the fact that women believed this method did not involve any costs, had no side effects, and was easy to practice. Other reasons in our study that prevented contraceptive use were dissatisfaction with sexual sensation, and husbands' unwillingness. Similarly, other investigators reported that most couples considered withdrawal due to health problems and side effects of modern methods [10,11]. A study from Turkey found that the reasons for using traditional methods and not effective methods among women were: wrong beliefs and fear of side effects (45.8%), unwillingness of men to use effective methods (37.5%), and cost of the methods (16.7%) [12]. In addition, the findings from present study indicated that preference of husbands, as a reason for using withdrawal, was relatively high (54.7%). A study from Turkey also showed that 31.2% of women used withdrawal due to the preference of their husbands [8]. Yet men are still not an important target group in most programs, and inadequate attention is paid to their role and their perspectives on issues of fertility control and they did not attend to health center for birth control. Even, in traditional societies such as Iran asking these questions from husbands is a very difficult task.

The present study showed that the main reasons for giving up oral contraceptives and intra uterine contraceptive device (IUCD) were side effects and health problems, while condoms stopped being used because of spouses dislike. This clearly suggests that two major reasons could be identified for withdrawal use: women-related and husband-related factors. With regard to women-related factors although we believe there should be a right for women, it seems that there is need to provide more support in order to help them to make a right decision. As far as husband-related factors involves, however, the issue of power and gender role might be relevant to discuss. This is consistent with the argument that men sometimes use withdrawal as a way to reinforce their decision-making and sexual control [13]. It has been suggested that gender-based power relations can have a direct effect on the ability of partners to acquire information relevant to their reproductive health, on their ability to make decisions related to their health, and on their ability to take action to protect or improve their health [14]. A study reported that side effects or health concerns accounted for a large portion of the relatively high first-year discontinuation rates for pills and injections (21% and 29%) [10]. The IUCD discontinuation rate was the lowest (%9) among all methods, compared with 38% for withdrawal and 56% for the pill [15]. In another study it was found that discontinuation rates for method-related reasons varied widely by method: IUCD was associated with the lowest probabilities of discontinuations (11% within 12 months, 30% within 4 years), followed by the pill (22% and 48%, respectively) and discontinuation rates were significantly higher for all other methods (condoms, withdrawal, fertility awareness methods and spermicides) [16]. Since modern contraceptive use may be associated with transient side effects, therefore for women who not prepared for these effects and not knowing where to go for follow-up and advice; discontinuation in the practice and development of fear regarding the use of modern methods might be expected.

The findings from current study showed that 72% of women could talk to their spouses about family planning. This suggests that the decision related to family planning is usually a jointly negotiated agreement by the couple, rather than a husband's imposition or a woman's choice alone (see Table 4). A study from Turkey reported that in 86 to 88% of cases the couple jointly made their contraceptive choices [17]. In general it is argued that reproductive decision-making is typically a jointly and co-operatively negotiation process by couples [18].

The scientific assertion that withdrawal has a relatively high failure rate is based on reports from a small number of studies, primarily conducted in North America, and with small sample sizes that may not be representative [19,20]. In one study among typical withdrawal users about 19% failed during the first year [21]. In another study, it was found that 48.6% unplanned pregnancies occurred while the couples were practicing withdrawal [8]. Prevention of unintended pregnancy is a significant public health issue and should be focus of health policies as it was the focus of healthy people 2010 in the USA [22].

Data from Iranian Demographic Health Survey in year 2000 indicated that about one-third of pregnancies were unintended [5]. Results from the present study showed that 37.2% of unwanted pregnancies occurred when the couples were practicing withdrawal and 62.8% were related to other reasons. Perhaps these pregnancies might lead to abortion. There are no reliable data on abortion in Iran as abortion is illegal except on occasions that the mother's life is in danger or in the case of fetal impairment [23]. A study of withdrawal users revealed that one out of four women reported that they terminated a pregnancy because it was unplanned [8]. The results of our study showed that 86 women experienced unwanted pregnancy while using different contraceptive methods. Of these 17 women reported that they terminated a pregnancy because it was unplanned (see Table 1). These women usually should pay a large amount of money for illegal abortions in illegal clinics; otherwise it could have serious consequences both for women and practitioners. It is argued that one reason for taking such a risk is that women do not want to have more children. For instance, a study from Turkey reported that women who had sufficient number of children preferred induced abortion instead of using an effective family planning method [24].

The current study found that 34% of women knew about emergency contraception while a study from Turkey revealed that only a few women (13.4%) knew about emergency contraception [8]. Emergency contraception has been defined as the use of a drug or a device to prevent pregnancy after intercourse and it has been shown to be safe and effective method to reduce the number of unwanted pregnancies [25]. Thus, there is a need to improve women's awareness about emergency contraception. The primary health care providers can play a major role in informing their patients about emergency contraception and it needs to become part of routine reproductive health counseling. To improve emergency contraception, awareness campaigns should be designed and implemented.

### Limitations

This study has some limitations. First, the study was carried out in one district of Tehran, and thus the findings cannot be generalized to withdrawal users who live in Iran. Secondly, the sample size was small. In addition the study was limited to women. Knowledge and attitudes of men need to be considered as well if we hope to make changes in the use of contraceptives in this population. However, this is the first study that investigated the experiences of and attitudes toward contraceptive methods among women who were using the withdrawal method in Tehran, Iran.

## Conclusion

The study results suggest that withdrawal users carry misconceptions about modern contraception and exaggerate its related health problems. The results provide insight into the complexity of the underlying decision-making processes and suggest that multidimensional interventions may needed to reduce the rate of unintended pregnancy. Contraception counseling should provide women with accurate information about contraception, and address any misconceptions women may have about the safety of various contraception methods. It is important to discuss the positive aspects of contraception during counseling so that women feel that their health will benefit more than be adversely affected. These findings might be essential for making evidence-based policy decisions, and for planning, monitoring and evaluating reproductive health programs in Iran and elsewhere.

## Competing interests

The authors declare that they have no competing interests.

## Authors' contributions

PR was the main investigator, designed the study, collected the data, performed analysis and wrote the first draft. AH supervised the study. FA, and AK, were the study advisor. ZGh helped the main investigator to finalize the research project and analyzing the data. AM was honorary advisor, contributed to the analysis, critically evaluated the paper, and responded to reviewers' comments, and provided the final draft. All authors read and approved the final revision of the manuscript.

## Pre-publication history

The pre-publication history for this paper can be accessed here:

http://www.biomedcentral.com/1471-2458/10/779/prepub

## Supplementary Material

Additional file 1 **Withdrawal Questionnaire (WQ)**. This file contains the questionnaire that was used in the study. It consists of three sections: Demographic & Reproductive Health Status; Reasons for Using Withdrawal; Experiences of and Attitudes toward Contraceptive Methods. Click here for file
