# Supplementary material for: Withdrawal users' experiences of and attitudes to contraceptive methods: a study from Eastern district of Tehran, Iran
Source: BMC Public Health. 2010 Dec 22;10:779. doi: 10.1186/1471-2458-10-779 (PMC3016392; doi:10.1186/1471-2458-10-779)
Supplement: Additional file 1 — Withdrawal Questionnaire (WQ). This file contains the questionnaire that was used in the study. It consists of three sections: Demographic & Reproductive Health Status; Reasons for Using Withdrawal; Experiences of and Attitudes toward Contraceptive Methods. [file 1471-2458-10-779-S1.DOC]

**Withdrawal Questionnaire**

**Parvin Rahnama et al. (2010)**

Section 1: Demographic & Reproductive Status

| **1. How old are you:** ……………..**years.** | | | | | |
| --- | --- | --- | --- | --- | --- |
| **2. Education:** | | | | | |
| Illiterate □ | Primary □ | | Secondary □ | | Higher □ |
| **3. Employment status** | | | | | |
| Housewife □ | Employed □ | | | | |
| **4. Duration of marriage (years):** …..….….. | | | | | |
| **5. Number of children:** …………….. | | | | | |
| **6. Have you had at least one unwanted pregnancy?** | | | | | |
| Yes □ | No□ | | | | |
| **7. If you have had unwanted pregnancy, have you used a contraceptive method?** | | | | | |
| Yes □ | No □ | | | | |
| **8. If yes, please indicate the method:** | | | | | |
| Withdrawal □ | OCP* □ | IUCD**□ | | Condom □ | Injection □ |
| **9. What was the outcome?** | | | | | |
| Delivery □ | Spontaneous abortion □ | | | Induce abortion □ | |

Section 2: Reasons for Using Withdrawal

| **10. Please indicate your main reasons for using withdrawal:** | |
| --- | --- |
| 10-1. I use withdrawal because it does not involve any costs. | |
| Yes □ | No □ |
| 10-2. I use withdrawal because it has fewer side-effects. | |
| Yes □ | No □ |
| 10-3. I use withdrawal because it is easier to use. | |
| Yes □ | No □ |
| 10-4. I use withdrawal because there is no need for counseling with a health care provider. | |
| Yes □ | No □ |
| 10-5. I use withdrawal because of my husband's preference. | |
| Yes □ | No □ |
| 10-6. I use withdrawal because I fear using other methods. | |
| Yes □ | No □ |
| 10-7. I use withdrawal because a health care provider suggested. | |
| Yes □ | No □ |
| **11. For how long do you practice withdrawal?** ……….…**years** | |

Section 3: Experiences of and Attitudes toward Contraceptive Methods

| **12: Have you used a modern contraceptive method before withdrawal method?** | | | | | | | | | | | | | | | | | | | | |
| --- | --- | --- | --- | --- | --- | --- | --- | --- | --- | --- | --- | --- | --- | --- | --- | --- | --- | --- | --- | --- |
| Yes □ | | No □ | | | | | | | | | | | | | | | | | | |
| **13. If yes, please indicate the method:** | | | | | | | | | | | | | | | | | | | | |
| OCP □ | | IUCD □ | | | | Condom □ | | | | | | | Injection □ | | | | | | |  |
| **14. Why did you stop using OCP?** | | | | | | | | | | | | | | | | | | | | |
| Because of its side effects □ | | | Thought might get pregnant □ | | | | | | | | | | | | Unwillingness of my husband □ | | | | | |
| Difficult to use □ | | | Willing to be pregnant □ | | | | | | | | | | | |  | | | | | |
| **15. Why did you stop using IUCD?** | | | | | | | | | | | | | | | | | | | | |
| Due to health problems (bleeding, pain, infection, etc.) □ | | | | | | | | | | | | | | | | Thought might get pregnant □ | | | | |
| Unwillingness of my husband □ | | | | | | | | | | | | Because it needs visit to health care services □ | | | | | | | | |
| Willing to be pregnant □ | | | | | | | | | |  | | | | | | | | | | |
| **16. Why did you stop using condom?** | | | | | | | | | | | | | | | | | | | | |
| Unwillingness of my husband □ | | | | | | | Thought might get pregnant □ | | | | | | | | | | | | | |
| Difficult to use □ | | | | | | | Access difficulty □ | | | | | | | | | |  | | | |
| **17. Why did you stop using injection?** | | | | | | | | | | | | | | | | | | | | |
| Thought might get pregnant □ | | | | | Due to health problems (amenorrhea, irregular bleeding, etc.) □ | | | | | | | | | | | | | | | |
| Unwillingness of my husband □ | | | | | | |  | | | | | | | | | | | | | |
| **18. What do you think about OCP, whether you have used it or not?** | | | | | | | | | | | | | | | | | | | | |
| I might become nervous (mood change) □ | | | | | | | | | | | I might get pregnant □ | | | | | | | | | |
| I might become infertile □ | | | | | | | | It might cause irregular bleeding □ | | | | | | | | | | | | |
| It might cause hirsutism □ | | | | It might cause nausea □ | | | | | | | | | | | It might cause weight gain □ | | | | | |
| **19. What do you think about IUCD, whether you have used it or not?** | | | | | | | | | | | | | | | | | | | | |
| It might cause irregular bleeding □ | | | | | | | | | It might cause infection □ | | | | | | | | | | It might cause pain □ | |
| It might cause translocation to other parts of the body □ | | | | | | | | | | | | | | I might get pregnant □ | | | | | | |
| **20. What do you think about condom, whether you have used it or not?** | | | | | | | | | | | | | | | | | | | | |
| I might get pregnant □ | | | | | Husband’s dislike □ | | | | | | | | | It might cause sexual dissatisfaction □ | | | | | | |
| Difficult to use □ | | | | | |  | | | | | | | | | | | | | | |
| **21. What do you think about injection, whether you have used it or not?** | | | | | | | | | | | | | | | | | | | | |
| It might cause irregular bleeding □ | | | | | | | | | | | | | | | | | | It might cause amenorrhea □ | | |
| It might cause weight gain □ | | | | | | I might get pregnant □ | | | | | | | | | | | |  | | |
| **22. Are you pleased with withdrawal method?** | | | | | | | | | | | | | | | | | | | | |
| Yes □ | | No □ | | | | | | | | | | | | | | | | | | |
| **23. Is your husband pleased with withdrawal method?** | | | | | | | | | | | | | | | | | | | | |
| Yes □ | | No. □ | | | | I do not know □ | | | | | | | | | | | | | | |
| **24. Did withdrawal method decrease your sexual enjoyment?** | | | | | | | | | | | | | | | | | | | | |
| Yes □ | No □ | | | | | I do not know □ | | | | | | | | | | | | | | |
| **25. Did withdrawal method decrease your husband's sexual enjoyment?** | | | | | | | | | | | | | | | | | | | | |
| Yes □ | No □ | | | | | I do not know □ | | | | | | | | | | | | | | |
| **26. Do you share your ideas about family planning with your husband?** | | | | | | | | | | | | | | | | | | | | |
| Yes □ | No □ | | | | | | | | | | | | | | | | | | | |
| **27. Who makes the decision about using contraceptives in your family?** | | | | | | | | | | | | | | | | | | | | |
| Myself □ | My husband □ | | | | | | | | | Myself and my husband together □ | | | | | | | | | | |
| **28. Are you worried to become pregnant while using withdrawal method?** | | | | | | | | | | | | | | | | | | | | |
| Yes □ | No □ | | | | | | | | | | | | | | | | | | | |
| **29. Are you familiar with emergency contraception?** *** | | | | | | | | | | | | | | | | | | | | |
| Yes □ | No□ | | | | | | | | | | | | | | | | | | | |
| **30. Do you know your period of ovulation time?** *** | | | | | | | | | | | | | | | | | | | | |
| Yes □ | No □ | | | | | | | | | | | | | | | | | | | |
| **31. Do you use condom during your period of ovulation time?** | | | | | | | | | | | | | | | | | | | | |
| Yes □ | | No □ | | | | | | | | | | | | | | | | | | |

**Thank you for your cooperation**

* OCP: oral contraceptive pill

** IUCD: intrauterine contraceptive device

*** If women responded correctly, then yes was considered.
